# Focus on localized laryngeal amyloidosis: management of five cases

**DOI:** 10.1515/med-2020-0400

**Published:** 2020-04-20

**Authors:** Massimo Mesolella, Gerardo Petruzzi, Sarah Buono, Grazia Salerno, Francesco Antonio Salzano, Giuseppe Di Lorenzo, Gaetano Motta

**Affiliations:** Department of Neurosciences, Reproductive and Odontostomatological Sciences, 80121 Naples, Italy; Department of Medicine, Surgery and Odontology, “Scuola Medica Salernitana” University of Salerno, Salerno, Italy; Department of Mental and Physical Health and Preventive Medicine, Head and Neck Surgery Unit, University of Campania “Luigi Vanvitelli”, Naples, Italy

## Abstract

Amyloidosis is a group of idiopathic clinical syndromes caused by the deposition of insoluble fibrillar proteins (amyloid) in the extracellular matrix of organs and tissues. These deposits disrupt the function of the target organ. Amyloidosis can manifest as a systemic disease or a single-organ involvement (local form). Its etiology still remains unclear. Deposits of amyloid in the larynx are rare, accounting for between 0.2 and 1.2% of benign tumors of the larynx. In this retrospective study, we report the clinical aspects, diagnosis, treatment and follow-up of five female patients with localized laryngeal amyloidosis without systemic involvement. The patients were all treated successfully using microlaryngoscopy with CO_2_ laser or cold instruments. Prognosis is excellent; however, appropriate follow-up is an important part of the long-term management of this disease in order to prevent and control the possibility of local recurrence.

## 1 Introduction

The term “amyloid” (*amylon*, in Greek) was coined by the pathologist Virchow, in 1854, to describe the deposits of a substance that he erroneously identified with starch [1,2].

Amyloids are formed by the assembly of an anomalous and heterogeneous group of protein precursors that take on a β-folded secondary structure and form fibrillar deposits (95%) in association with other nonprotein substances (5%).

The accumulation of amyloids in the interstitial spaces of organs and tissues disrupts the architecture and functionality of the latter. Fibrils may deposit locally or may involve every organ systemically; the current classification distinguishes a systemic form and a localized one, in which the deposits affect a single organ without a systemic disease [3].

Until today, at least 27 types of amyloidoses have been identified, based on the different proteins that form in vivo amyloid fibrils. According to this classification, a lettering system describes different forms: the amyloid protein is designated with the capital letter A, followed by a suffix that specifies the nature of the protein substance or the precursor protein name. For example, the abbreviation AL designates amyloidosis derived by the light chains of immunoglobulins [3,4].

The systemic variant generally implies a poor prognosis with a clinical picture that evolves more or less rapidly toward exitus. On the other hand, localized amyloidosis is a rare condition that can be observed in every organ and presents a favorable evolution in most cases [4].

The first case of laryngeal amyloidosis (LA) reported in the literature was published by Burow and Neumann in 1875 and referred to as an occasional autopsy finding [2]. The larynx is the organ most frequently affected in isolated localization of the head–neck region, and LA should be considered extremely rare as it accounts for just 0.2–1.2% of all benign neoplastic manifestations of this organ. The larynx can be involved at all levels; a careful review of the literature shows a different distribution of its localization that affects, in descending order, the false cords, the aryepiglottic fold and the subglottic region [5].

A greater incidence in men has been reported, with a 3 : 1 male–female ratio, especially between the fifth and the sixth decade of life [6].

The symptomatology of LA is nonspecific and is characterized in most cases by hoarseness, dysphonia, foreign body sensation, dysphagia or dyspnea.

The “gold standard” for the diagnosis of amyloidosis is based upon histological examination. Histology of the specimen using routine hematoxylin and eosin staining shows amyloid as the eosinophilic extracellular infiltrate, sparing the overlying epithelium. Further staining with Congo red shows a characteristic apple green birefringence and dichroism under a polarizing microscope. Staining with methyl violet discloses metachromatic pink-violet staining.

Regarding clinical objectivity, LA generally results in one or more areas of swelling, located in the submucosa, covered by apparently healthy mucosa. This clinical aspect, so common in laryngeal infections, leads to important problems of differential diagnosis between benign diseases (such as congenital cysts and laryngocele) and malignant ones (such as infiltrating carcinoma, chondroma or lymphoma) [5,6].

The treatments available depend on the site and size, as well as on the general condition, age and personal preference of the patient; before starting any therapy, it is essential to have an unequivocal documentation of the type of amyloidosis [7].

The low prevalence of this pathology poses in the first place many diagnostic difficulties, and there is still no clear unequivocal treatment and management modality.

This study aims to report the surgical experiences related to our cases, discuss the indications of the techniques used and summarize our report in comparison to others in the literature.

## 2 Materials and methods

Five patients with LA were treated and followed in the Otorhinolaryngology Unit of the “Federico II” University of Naples from 1982 to 2018 (Table 1).

**Table 1:** Patient summary: demographics, disease locations, symptom, imaging and treatment

Patient summary	Case 1	Case 2	Case 3	Case 4	Case 5
Age (years)	14	21	34	47	38
Sex	Female	Female	Female	Female	Female
Disease location	Left false cord	Right false cord and aryepiglottic fold	Subglottic	Diffuse larynx involvement	Left false cord
Symptoms	Dysphonia	Dysphonia, vocal fatigue	Dysphonia, stridor	Dysphonia, dyspnea	Dysphonia
Systematic disease	No	No	No	No	No
Imaging	CT	CT	CT	CT	MRI
Operation	Laser CO_2_	Laser CO_2_	Laser CO_2_	Laser CO_2_	Cold technique

The five subjects studied are all female, in the age range between 14 and 47 years.

All patients underwent radiographic examination (CT in the first four cases and MRI in the fifth case) to define the localization and extension of laryngeal lesions.

In collaboration with the pathologists and hematologists of our university, the simultaneous presence of systemic amyloidosis was excluded. The work-up involved blood tests, echocardiogram, evaluation of renal function, hepatic function and biopsy of periumbilical fat and bone marrow. The type of amyloid was defined by using the immunogold labeling technique using electron microscopy.

The exeresis is performed under general anesthesia using direct laryngoscopy. We tried to implement the surgical excision en bloc with adjacent safe tissues. In the first 6 months, the patients underwent monthly laryngoscopic check-ups and surgical evaluation in order to remove fibrin deposits or avoid scar adhesions or to perform biopsies; subsequently, bimonthly endoscopic check-ups were performed for the remainder of the first year and for the second year, then six-monthly for 2 years and yearly from the fifth year [8].

### 2.1 Case 1

A 14-year-old female patient was admitted to the hospital with dysphonia that had arisen about 1 year ago and gradually progressed. Laryngeal examination revealed the presence of a circumscribed mass, arising from the left false vocal cord. Using endoscopy, with the CO_2_ laser, this lesion was removed; the histological examination of the surgical specimen highlighted the presence of a circumscribed amyloidosis nucleus.

After about a month, a microlaryngoscopy check was performed: some biopsies were taken, and the histological examination excluded the presence of new amyloidosis manifestations.

There has been no evidence of recurrence during follow-up, with an optimal vocal function outcome.

### 2.2 Case 2

A 21-year-old female patient was hospitalized for dysphonia with vocal fatigue that had arisen about 2 years ago, which had progressively worsened. Clinical objectivity demonstrated the presence of a mass involving the right false vocal cord and the adjacent aryepiglottic fold.

The lesions were removed using CO_2_ laser, and the typical pattern of LA was identified histologically. Approximately 1 month after the operation, a mass growing in the right false vocal cord extending to the adjacent portion of the epiglottis was identified. With the appearance of dysphonia, a new endoscopic resection using the CO_2_ laser was performed and a well-circumscribed submucosal amyloid deposit, surrounded by granulation tissue, was documented by the pathologist.

After several years, the patient no longer showed signs of recurrence or functional damage.

### 2.3 Case 3

A 34-year-old female patient was admitted to the hospital with a 1-year history of dysphonia and stridor; laryngeal examination documented the presence of two “pads” covered with mucosa of normal appearance, located in the subglottic region and extending to the anterior commissure. Using endoscopy, with the CO_2_ laser, the lesion was removed en bloc; the histological examination revealed the presence of submucosal deposits of amyloid, surrounded by healthy tissue.

Fibrin deposits were removed 1 month after first operation during an endoscopic check-up for dyspnea and no signs of recurrence were documented. An excellent functional result persists.

### 2.4 Case 4

A 47-year-old female patient was admitted to the hospital for dysphonia and dyspnea that had appeared about 2 years ago. With fiberoptic laryngoscopy, various submucosal tumefactions were observed, involving the supraglottic, glottic and subglottic regions, especially on the right. A biopsy was performed, and no tumor was found. Using endoscopy, with the CO_2_ laser, the lesions were removed. Histological examination highlighted the presence of extensive accumulations of amyloid substance even on the excision margins.

New symptomatic and large amyloid lesions were removed three times from both the vocal folds and at the level of the epiglottic pedicle. Finally, endoscopic CO_2_ laser treatment was performed in order to vaporize the edematous flap in the left arytenoid region and resect a synechia at the level of the anterior commissure 2 years after the first surgical procedures.

The patient remains with dysphonia and glottic insufficiency, but there was no evidence of recurrence.

### 2.5 Case 5

A 38-year-old female patient sought our attention for the onset of insidious dysphonia for about 4 years. She underwent surgery of the vocal cords using direct microlaryngoscopy for dysphonia, in another institute; the histological examination showed the presence of an amyloid substance but was not supported by the systemic study.

**Figure 1:**
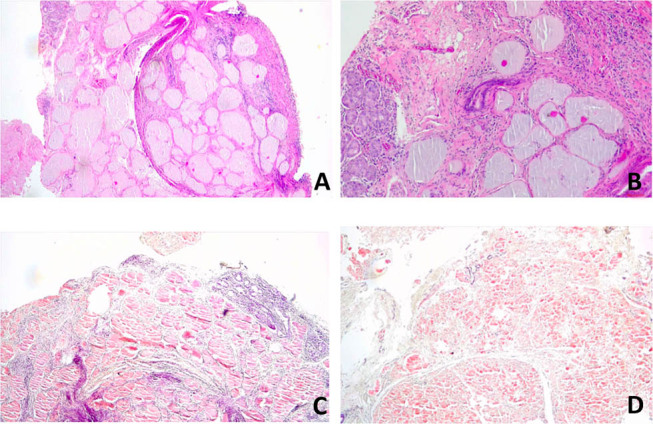
Histological preparation. (A) Excisional biopsy of the left vocal cord reveals a deposit of amorphous eosinophilic material in the tissue, consisting of amyloid (hematoxylin–eosin stain). Magnification 4×. (B) Detailed image of amyloid deposits. Magnification 10×. (C and D) Deposits are highlighted on Congo red stain. Magnification 10×.

The fiberoptic laryngoscopy demonstrated a red subepithelial mass on the left false vocal cord extending to the laryngeal ventricle. The neoformation was removed using microlaryngoscopy by the cold steel technique and subjected to further histological examination, which confirmed the diagnosis of amyloidosis (Figure 1). Systemic amyloidosis was excluded with clinical and laboratory investigations.

**Figure 2:**
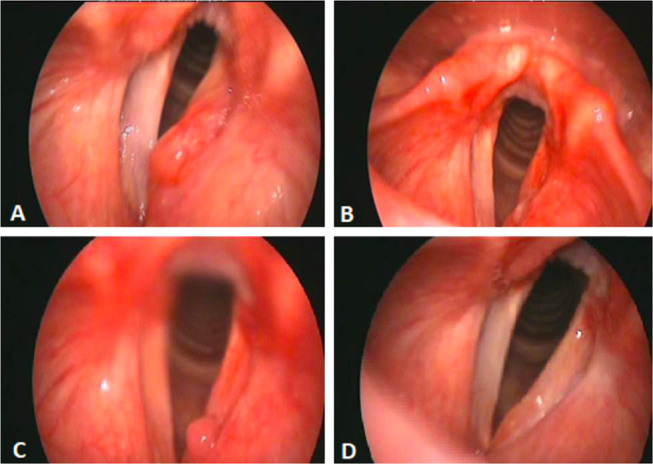
Case 5: endoscopic image before (A) and after (B) the surgical excision. A small formation in correspondence of the surgical excision at 4-month laryngeal control (C). Last follow-up examination (D).

**Figure 3:**
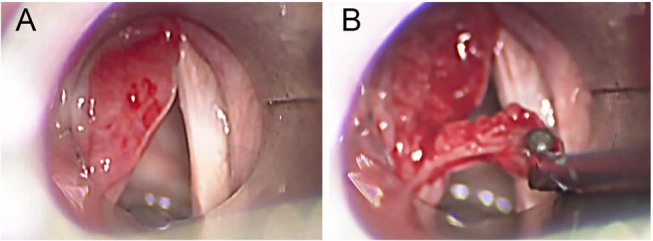
Intraoperative images: before (A) and during (B) the surgical excision.


After 4 months of the surgical operation, an endoscopic control showed an asymptomatic small formation in correspondence with the surgical excision. In order to identify its nature, this lesion was closely monitored. In the successive laryngeal control, this mass had highly reduced itself and then disappeared. To date, after 1 year of follow-up, no other signs of recurrence appeared, and the function of the vocal cord has been excellent (Figures 2 and 3).

**Ethical approval:** This research related to human use complied with all the relevant national regulations and institutional policies and was conducted in accordance with the tenets of the Declaration of Helsinki and approved by the institutional review board or equivalent committees of the authors’ institutes.
**Informed consent:** Informed consent was obtained from all individuals included in this study.

## 3 Results

In the aforementioned five cases, there are different anatomical and clinical pictures with different surgical implications. All patients showed symptoms before undergoing surgical treatment. In cases 1, 2, 3 and 5, endoscopic treatment demonstrated its indisputable validity; in case 4, this surgical approach led to problems and needed further interventions. This depends on the extent of the amyloidosis lesions; external conservative interventions, on the other hand, would not have been less complex or invasive. In case 5, the use of the cold technique is related to the small extension: it allowed a more rapid patient outcome, short hospitalization time and the possibility of controls in the absence of postoperative fibrin.

Amyloid lesions generally have indistinct margins that are not easy to identify during surgery, and therefore the need for surgical revisions cannot be excluded a priori. Narrow-band imaging endoscopy (NBI) procedure, tested in the last case, cannot help the surgical choice. Currently, the cases have all perfectly recovered from the pathological process and present excellent functional results.

## 4 Discussion

The clinical and surgical experiences reported in the literature on LA consist of case reports and small case series. To date, several treatment options have been reported.

In our cases, there is a clear prevalence among the female sex. This epidemiological observation, verified by appropriate methods, would perhaps open a new path in the discovery of the etiopathogenesis of the pathology, which is still unknown.

Regarding the site, according to the literature, in our cases the supraglottic areas are the most frequently affected ones, in particular the false vocal cords (cases 1, 2, 4 and 5) [3,4,5,6,7,8].

Imaging techniques such as computed tomography and magnetic resonance play a fundamental role in determining the extension of the lesion or characteristic infiltration signs. MRI could play a superior role for this purpose. The characteristic signal of amyloidosis reveals an intensity equal to that of the skeletal muscles: intermediate *T*1-weighted signal intensity and low *T*2-weighted signal intensity. This is an important point of differentiation because the muscle is a simple reference and tumors do not appear in this way [9].

The only treatment for symptomatic LA is surgical. The treatment goal is to offer the patient a long disease-free interval with functional larynx preservation. There are conflicting views on the type, modality and timing of surgery [3].

The goal of localized LA treatment is to offer to the patient as long a disease-free interval as possible with voice preservation and normal breathing.

Some authors support the need for an external approach to completely dominate the lesion and promote its radical excision; others are instead convinced advocates of endoscopic surgery [3,10,11,12].

There are multiple endoscopic options (suspended microlaryngoscopy and tumor removal with CO_2_ laser or cold instruments). In our experience, the technique choice depends on the location and extent of the lesions. The use of endoscopic surgery is demonstrated herein, underlining the possibility of obtaining a definitive clinical recovery in the five cases studied. Benefit of this surgery are radical excision of the tumor with a reduced trauma, excellent postoperative functionality results, short hospitalization and low socio-health costs.

Regarding follow-up, the literature is still unclear; therefore, the need for careful and regular follow-up is emphasized [13].

In fact, although the prognosis of LA is generally good, the probability of recurrent or residual disease is significant and death due to respiratory failure has been reported [14].

In our opinion, this probably depends on an imprecise clinical evaluation of the peripheral margins of the lesions [15]. Unfortunately, there are no specific macroscopic characteristics that make it easy to identify the infiltration margins in laryngoscopy or during surgical excision [16].

For our patients, we performed monthly check-ups for the first 6 months, bimonthly until the end of the second year, then six-monthly for two more years and yearly from the fifth year onwards (Table 2).

**Table 2:** Follow-up scheme

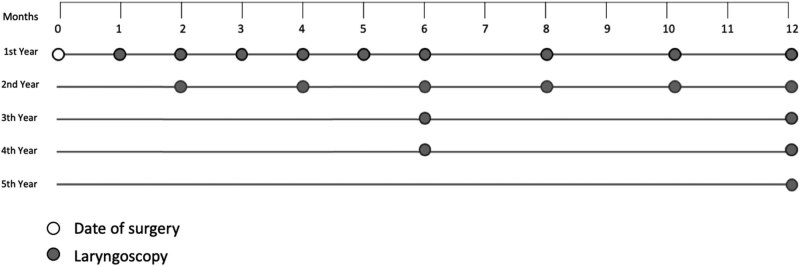

Therefore, a laryngological follow-up period of at least 10 years after the last surgery is recommended: a later progression remains possible.

## 5 Conclusion

LA is a rare disease and remains a not completely understood pathology. LA is prevalent in the male sex and affects the supraglottic regions, in particular the false vocal cords and the ventricle. Concerning epidemiology, other studies may confirm our data: probably, systemic amyloidosis is a prerogative of the male sex, whereas the laryngeal form is more frequent in the female sex. The first-choice treatment is surgery; in our opinion, the main aim is to guarantee organ functionality: the options are local endoscopic procedures such as CO_2_ laser or cold steel technique.

A fundamental part of the care pathway should be an adequate follow-up. We recommend monthly endoscopic check-ups for the first 6 months, then bimonthly until the end of the second year, then six-monthly for 2 years and yearly from the fifth year. Long-term follow-up should always be performed.
